# Non-traumatic chest pain in patients presenting to an urban emergency Department in sub Saharan Africa: a prospective cohort study in Tanzania

**DOI:** 10.1186/s12872-019-1133-0

**Published:** 2019-06-28

**Authors:** Amour S. Mohamed, Hendry R. Sawe, Biita Muhanuzi, Nafsa R. Marombwa, Kilalo Mjema, Ellen J. Weber

**Affiliations:** 1 Emergency Medicine Department, Muhimbili University of Health and Allied Science, P.O. Box 65001, Dar es Salaam, Tanzania; 2 Emergency Medicine Department, Muhimbili National Hospital, Dar es Salaam, Tanzania; 3 Department of Emergency Medicine, University of California, San Francisco, CA USA

**Keywords:** Chest pain, Emergency department, Non-traumatic chest pain, Sub Sahara Africa, Tanzania, Acute coronary syndrome, Tuberculosis

## Abstract

**Background:**

Non-traumatic chest pain (NTCP) is a common reason for emergency department (ED) attendance in high-income countries, with the primary concern focused on life threatening cardiovascular diseases. There is general lack of data on aetiologies, diagnosis and management of NTPC in Sub Sahara African (SSA) countries. We aimed to describe evaluation, diagnosis and outcomes of adult patients presenting with NTCP to an urban ED in Tanzania.

**Method:**

This was a prospective observational cohort study of consecutive adult (≥18 years) patients presenting with non-traumatic chest pain to the Emergency Medicine Department (EMD) of Muhimbili National Hospital (MNH) in Dar es salaam from September 2017 to April 2018. Structured case report form was used to collected demographics, clinical presentation, investigations, diagnosis, and EMD disposition and in hospital mortality. We determined frequency of NTCP among our patients, aetiologies, 24-h and 7-day in-hospital mortality, and predictors for mortality.

**Results:**

We screened 29,495 adults attending EMD-MNH during the study and 389 (1.3%) presented with NTCP of these, 349 (90%) were enrolled. The median age was 45 (IQR 29–60) years and 177 (50.7%) were female. Overall, 69.1% patients received electrocardiography (ECG) in the EMD and 34.1% had a troponin test. Heart failure and pulmonary tuberculosis (PTB) were the leading hospital diagnoses (12.6% each), followed by chronic kidney disease (10%) and acute coronary syndrome (ACS) (9.6%). Total of 167 (48%) patients were admitted, and the 24-h and 7-day in-hospital mortality were 5 (3%) and 16 (9.6%) respectively. Univariate risk factors for mortality were a Glasgow Coma Scale of < 15 [RR = 3.4 (95%CI 3.2–23)], Acute Coronary Syndrome [RR = 5.7 (95% CI 1.7–11.8) and Troponin > 0.04 ng/ml [RR 2.9 (95%CI 1.2–7.3)]. Features distinguishing cardiovascular from other causes were: bradycardia [RR = 2.6 (95%CI 2.1–3.2)], heart beat awareness [RR = 2.3 (95%CI 1.7–3.2)] and history of diabetic mellitus [RR = 2.2 (95% CI 1.6–3.0)].

**Conclusion:**

In this ED of SSA country, heart failure and pulmonary tuberculosis were the leading causes of NCTP, and ACS was present in 9.6%. NTCP in this setting carries high mortality, and ACS was the leading risk factor for death. ED providers in SSA must increasingly consider cardiovascular causes of NTCP.

## Background

Globally, non-traumatic chest pain (NTCP) is a significant problem which affect about 20–40% of the general population in their lifetime [1, 2]. NTCP is common cause of visits to the emergency department (ED). It is a symptom with a broad differential diagnosis [3], including potentially lethal causes such as Acute Coronary Syndrome (ACS), aortic dissection, and pulmonary embolism [3, 4]. Thus, NTCP presents a high-risk diagnostic challenge in the ED. Detecting ACS is particularly important as one-third of ST elevation MI (STEMI) patients die within 24 h after ischemia onset [5] It has been reported that 46% of deaths in ED patients are caused by myocardial infarction and 27% are caused by other ischemic heart diseases [6, 7].

In high income countries (HICs) NTCP is the second leading cause of an ED visit after abdominal pain, the symptoms suggestive of myocardial infarction (MI) contributes up to 8–10% of ED visits yearly and cardiac disease is estimated to be the aetiology in 8–18% of all cases of chest pain presenting to the ED [1, 8, 9]. Recent evidence suggests that in the USA, 6% of NTCP patients in the ED have unrecognized important myocardial damage, and about 3% of MI patients are erroneously discharged, leading to higher mortality than those admitted [10–12].

There are few studies in Sub Saharan Africa (SSA) describing the aetiology and outcomes of patients with NTCP. The data that does exist suggests that the incidence of NTCP in acute care settings is much lower than in HIC’s (1.66%), respiratory diseases are the most common cause of NTCP, with pneumonia accounting for 24.4% of patients admitted in this patient population [13, 14], However, as the prevalence of hypertension and diabetes mellitus (DM) increases in these population, the potential for cardiovascular disease has recently gained attention.

Given the paucity of data, and the changing profile of risk factors in SSA, we aimed to determine the incidence, and describe medical evaluation and outcome of adult patients presenting with NTCP to the first full capacity ED in Tanzania. The findings will allow us to understand the aetiologies of NTCP in our population, helping to direct health care resources and establish protocols for evaluation and management.

## Methods

### Study design

This was a prospective observational cohort study of adult patients (≥18 years) who presented with NTCP in the emergency medicine department (EMD) of Muhimbili Hospital in Dar es Salaam, from September 2017 to April 2018.

### Study setting

Muhimbili National Hospital (MNH) is a tertiary referral hospital with a bed capacity of 1500 beds, and the main clinical training site for the Muhimbili University of Health and Allied Sciences (MUHAS). The EMD opened in 2010, and it is the site for the only emergency medicine residency training program in the country. The department is staffed by specialist emergency physicians, who provide clinical care, supervision and teaching to interns, registrars (generalists) and emergency medicine residents. The MNH-EMD receives high acuity patients from within Dar-es-Salaam and the surrounding regional and district hospitals; it served over 60,000 patients in 2016.

### Study protocol

A research assistant was present in the department at all times during the study period. All arriving patients were screened for the chief complaint of chest pain by a review of the electronic tracking board. Patients with the chief complaint of non-traumatic chest pain (chest pain unrelated to recent trauma) were approached for consent, and if agreed, enrolled in the study. A standardised case report form was used to document patient demographics, presenting complaints and duration, comorbidities, physical findings, investigations, consultation, and EMD management and disposition. All Electrocardiography were interpreted by ED providers who were attending a patient and consulted cardiology team, chest X rays were initially interpreted by ED providers and the interpretation were confirmed by the radiology report obtained in the hospital electronic medical record system after being reported by a radiologist. Admitted patients were followed up at 24 h and 7 days for mortality and final hospital/discharge diagnosis. Data collection was done through interviewing patients and/or next of kin and reviewing the patient’s records in the EMD while the patient was still present, and later the hospital patient’s medical records for outcomes.

### Data analysis

The data was transferred into a research electronic data capture (REDCap) application and then imported into statistical package for social science (SPSS) (version 23.0.0, IBM LTD, Carolina, USA). Patient characteristics were reported as mean and standard deviation (SD), or median and interquartile range (IQR) for continuous variables and number/proportion for categorical variables. Univariate analysis was used to calculate the relationship between the clinical predictors (abnormal vitals and abnormal investigation results, final hospital diagnosis) and hospital mortality. Relative risk with 95% Confidence interval (95%CI) and Chi-Squire (X^2^) test was used to test the significance of variables association. A *p* value of < 0.05 was considered statistically significant.

## Results

**Fig. 1:**
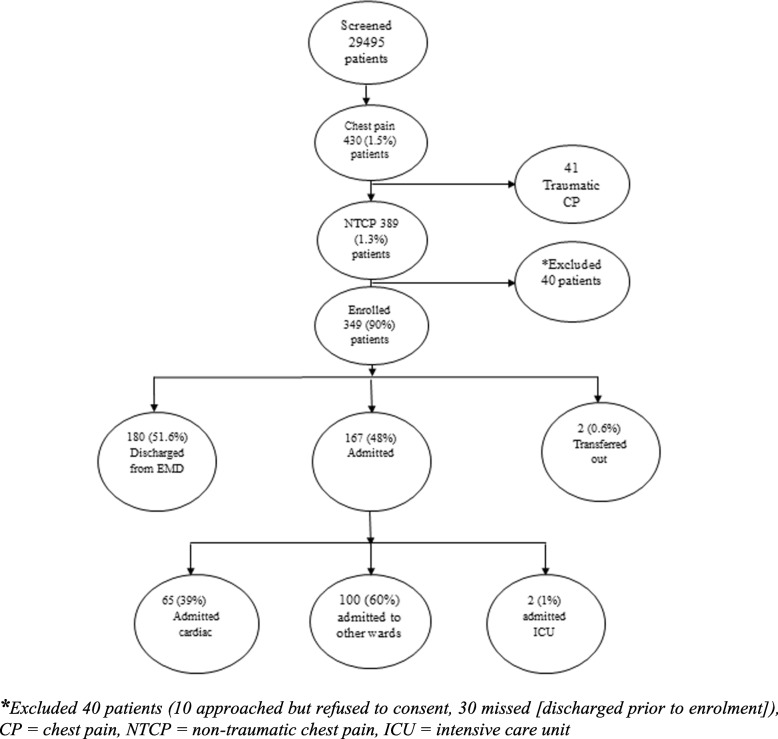
Screening, enrollment and disposition of adults patients presented to the EMD MNH with the primary complaint of non-traumatic chest pain

We screened 29,495 adults patients 18 years and above who presented to the EMD during the study period; among them 430 (1.5%) presented with the complaint of chest pain (traumatic and non-traumatic), and of those, 389 (1.3%) presented with non-traumatic chest pain. Ten patients refused participation and 30 were missed (discharged prior to enrolment). The remaining 349 consented to participate and were enrolled in the study (Fig. 1).

### Patient characteristics

Of the 349 patients enrolled, median age was 45 years, (IQR: 29–60) years, and 60% were under 50 years old. There were 177 (50.7%) females. 194 (55.6%) patients had at least one comorbidity; hypertension was most common. 43 (12.3%) arrived to the EMD by ambulance. For 181 (52%) patients this was the first episode of non-traumatic chest pain. The median duration of current illness was 7 days (IQR 2–30 days). Overall, 99 (28.4%) patients were referred from other peripheral hospitals. Overall, the most common reported associated symptoms were shortness of breath/difficulty in breathing (SOB/DIB) 31.2% *n* = 109, followed by cough 17.8% *n* = 62, heart beat awareness (10.6% *n* = 37. 38% *n* = 132 patients had increased respiratory rate, 27.4% *n* = 95 had tachycardia, 15.4% *n* = 54 had mean arterial pressure > 110mmhg and 8.9% *n* = 31 had hypoxia (Table 1).

**Table 1:** Characteristics of study patients

Variable	Number (%)
Overall	349 (100%)
Age (years), median (IQR)	45 (29–60)
Below 50 years	212 (60.7)
Above 50 years	137 (39.3)
Female Sex	177 (50.7)
Common comorbidity	194 (55.6)
Hypertension	82 (23.5)
Heart failure	48 (13.8)
Diabetes Mellitus	26 (7.4)
HIV infection	12 (3.5)
Chronic kidney disease	10 (2.9)
Sickle cell disease	9 (2.6)
Arrived by ambulance	43 (12.3)
Arrived by non-ambulance	306 (87.7)
Referral from peripheral hospitals	99 (28.4)
Self – referral	250 (71.6)
Associated symptoms	
Shortness/difficulty in breathing	109 (31.2)
Cough	62 (17.8)
Heart beat awareness	37 (10.6)
Chest tightness	32 (9.2)
Fever	29 (8.3)
Abnormal vital signs	
Tachypnea (RR > 20breaths/min at rest)	132 (38.0)
Tachycardia (HR > 100beats/min at rest)	95 (27.4)
High MAP (> 110mmhg)	54 (15.4)
Hypoxia (Spo2 < 94% in room air)	31 (8.9)
GCS < 15	20 (5.7)

*HIV* human Immunodeficiency virus, *RR* respiratory rate, *min* minutes, *HR* heart rate, *MAP* mean arterial pressure, *Spo2* saturation partial pressure of Oxygen, *GCS* Glasgow comma scale

### Patients outcomes

Among the 349 patients, 167 (48%; 95% CI 43–53%) patients were admitted, 180 (51.6%) were discharged home from the EMD, 2 (0.6%) were transferred to another hospital. The majority of admissions (57%) were over 50 years old (Table 2).

**Table 2:** Disposition from EMD and outcomes of admitted patients

	Overall *N* = 349, n /N (%)	Patients < 50 years *N* = 212 n /N (%)	Patients > 50 years *N* = 137 n /N (%)
Transferred out	2/349 (0.6)	–	2/137 (1.5)
Admission	167/349 (48)	89/212 (42)	78 /137 (57)
Admitted to wards	165/167 (99)	87/89 (98)	78/78 (100%)
Admitted to ICU	2/167 (1)	2/89 (2)	–
Mortality	*N* = 167	*N* = 89	*N* = 78
24-h mortality	5/167 (3)	3/89 (3.4)	2/78 (2.6)
7-day in-hospital mortality	16/167 (9.6)	7/89 (8.0)	9/78 (11.5)

Total of 65 (39%) of admitted patients were admitted to the cardiology service (Cardiology Institute,) Two (1.0%) were admitted to medical ICU and 100 (60%) were admitted to hospital wards according to presumed aetiology and presenting symptoms (Fig. 1).

The 24 h in-hospital mortality was 3% (*n* = 5) and the 7 day in-hospital mortality was 9.6% (*n* = 16), No patient died at EMD (Table 2).

### ED evaluation

**Fig. 2:**
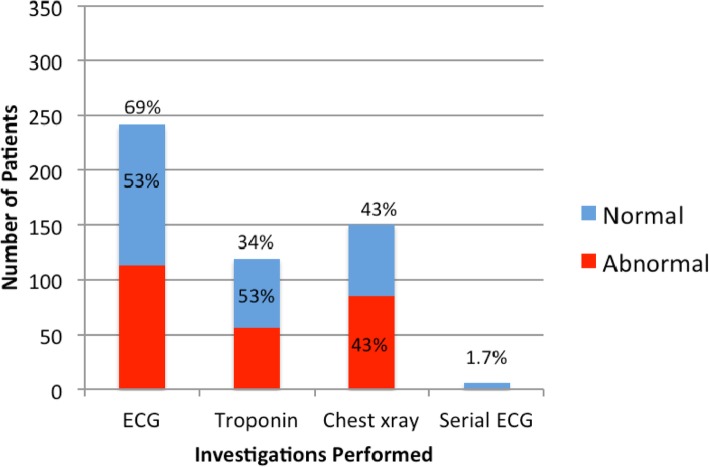
EMD most frequent investigations performed and result

Among all 349 patients, 69% had an initial ECG, 47% of them were abnormal; 34% had troponin sent, 47% of them were > 0.04 ng/ml; and 43% had chest X-ray done, 43% of them were abnormal (Fig. 2).

### Final hospital diagnosis and mortality

The most frequent final hospital diagnoses were heart failure and pulmonary tuberculosis (PTB) 12.6% *n* = 21 each, followed by chronic kidney disease 10% *n* = 17 and Acute Coronary Syndrome (ACS) 9.6%, *n* = 16. Only the diagnosis of ACS conveyed an increased risk of death. (Table 3) Significant risk factors for mortality were having ACS (RR = 5.7), GCS < 15 (RR = 3.4), Troponin > 0.04 ng/ml (RR-2.9) and difficulty in breathing (RR = 2.8) (Table 4). Patients who had cardiovascular disease (CVD) were more likely to have heart rate < 60beats per minute (RR 2.6), heart beat awareness (RR 2.3), history of diabetic mellitus (RR 2.2), history of heart disease (RR 2.1) and older age > 50 years (RR 2.1) (Table 5).

**Table 3:** Final hospital diagnoses

No	Final Diagnosis	Overall N = 167 n (%)	Alive *N* = 151 n (%)	Died *N* = 16 n (%)	Relative Risk (95%CI)
1.	Heart failure	21 (12.6)	17 (11.3)	4 (25)	2.3 (0.8–6.5)
2.	Pulmonary Tuberculosis	21 (12.6)	21 (14)	–	0.2 (0.01–3.3)
3.	Chronic kidney disease	17 (10)	14 (9.3)	3 (18.8)	2.0 (0.6–6.4)
4.	Acute coronary syndrome	16 (9.6)	10 (6.6)	6 (37.5)	5.7 (2.4–13.5)
5.	Pneumonia	13 (7.8)	13 (8.6)	–	0.3 (0.02–5.3)
6.	Lungs Cancer	9 (5.4)	7 (4.6)	2 (12.5)	2.5 (0.7–9.4)
7.	PUD/GERD/Gastritis	9 (5.4)	9 (6)		0.5 (0.03–7.5)
8.	COPD	6 (3.6)	6 (4)	–	0.7 (0.05–10.5)
9.	Cardiomyopathy	6 (3.6)	6 (4)	–	0.7 (0.05–10.5)
10.	Hypertensive heart disease	6 (3.6)	5 (3.3)	1 (6.3)	1.8 (0.3–11.4)
11.	Others	43 (26)	43 (28.5)	–	

*COPD* chronic obstructive pulmonary disease, *PUD* peptic ulcer disease, *GERD* gastro-esophageal reflux disease

**Table 4:** Risk factors of in-hospital mortality at 7 days (*N* = 167)

Variable	Number n	Alive *N* = 151	Dead *N* = 16	Relative risk (95% CI)
Age > 50 years	79	71 (90)	8 (10)	1.1 (0.4–2.8)
History of Diabetic mellitus	16	14 (87.5)	2 (12.5)	1.35 (0.3–5.4)
Arrived by ambulance	43	39 (90.7)	4 (9.3))	0.96 (0.3–2.8)
Difficulty in breathing	73	62 (85)	11 (15)	2.8 (1.03–7.8)
GCS < 15	15	11 (73.3)	4 (26.7)	3.4 (1.2–9.1)
Abnormal ECG	72	62 (86)	10 (14)	2.2 (0.8–5.8)
Troponin > 0.04 ng/ml	35	28 (80)	7 (20)	2.9 (1.2–7.3)
Heart failure	21	17 (81)	4 (19)	2.3 (0.8–6.5)
Pulmonary Tuberculosis	21	21 (100)	–	0.2 (0.01–3.3)
Chronic kidney disease	17	14 (82)	3 (18)	2.0 (0.6–6.4)
Acute coronary Syndrome	16	10 (62.5)	6 (37.5)	5.7 (2.4–13.5)

*DIB* difficulty in breathing, *GCS* Glasgow coma scale, *ECG* electrocardiography, *CI* confidence interval

**Table 5:** Risk factor for cardiovascular disease/diagnosis (CVD)

Risk factor/Presentation	Number n	CVD *N* = 65	Non CVD *N* = 102	RR for CVD (95% CI)
Age > 50 years	78	44 (56)	34 (44)	2.1 (1.4–3.1)
Male	81	37 (45.7)	44 (54.3)	1.2 (0.9–1.8)
History of Diabetic Mellitus	16	13 (81.3)	3 (18.7)	2.2 (1.6–3.0)
History of heart disease	31	22 (71)	9 (29)	2.1 (1.5–2.8)
History of Hypertension	47	28 (59.5)	19 (40.5)	2.0 (1.4–2.8)
Difficulty in breathing	72	38 (52.8)	34 (47.2)	1.5 (1.1–2.2)
Heart beat awareness	16	13 (81.3)	3 (18.7)	2.3 (1.7–3.2)
Heart Rate < 60 beats/min	03	3 (100%)	–	2.6 (2.1–3.2)
MAP >110mmhg	54	32 (59)	22 (41)	2.0 (1.4–3.0)
Initial SBP ≥ 140/DBP ≥ 90mmhg	45	23 (51)	22 (49)	1.4 (0.9–6-2.)
Heart rate > 100 beats/min	63	23 (36.5)	40 (63.5)	0.8 (0.6–1.3)
Hypoxia (SPO2 < 94% RA)	26	9 (34.6)	17 (65.4)	0.8 (0.5–1.4)

*MAP* mean arterial pressure, *SBP* systolic blood pressure, *DBP* diastolic blood pressure, *mmhg* millimetre of mercury, *SPO2* saturation partial pressure of oxygen, *RA* room air, *CI* confidence interval

## Discussion

In this study, we found that 1.3% of adult patients presenting to the EMD-MNH had a primary complaint of non-traumatic chest pain (NTCP). Nearly half of patients were admitted, and mortality was 9.6%, higher than in many high income countries (HICs). Most patients presenting with NTCP were young, while the majority of those admitted were > 50 years. Strengths of this study were that patients were enrolled prospectively and consecutively, with 24-h enrolment during the study period. No admitted patients were lost to follow up and aetiology was based on hospital rather than ED diagnoses. MNH is a referral hospital, and patients are referred to the ED from all over the country.

The frequency of chest pain complaints in our study is lower than that in HICs; studies from EDs in the United states, Europe and United kingdom report that from 2 to 5% of patients present with NTCP [5, 15–17]. However, our data are consistent with a study done in Pretoria, South Africa, where 1.66% of patients presented with NTCP [14].

Our findings are also in contrast to studies done in HICs where the majority of patients presenting to the EMD with NTCP were elderly [18]. In our study, the median age was 45 years. Studies in low and middle income countries (LMICs) have reported a similar age range [13]. This can be partly explained by the differences in etiologies of NTCP, in our study, pulmonary TB was one of the two leading causes of NTCP among those admitted, and patients of any age are at risk, while TB is far less common in HICs. Additionally, hypertension and hypertensive heart disease have an early age of onset in our population, and many patients do not receive timely preventive treatment [19, 20].

Previous studies in LMICs reported that respiratory disease is the leading cause of NTCP presentations to emergency departments [14]. However, in this study, heart failure and PTB were equally common as the primary etiologies, followed by CKD and then ACS. The current study also highlights the prevalence of cardiovascular risk factors and the increasing prominence of cardiovascular causes for NTCP in LMICs [3, 21] If one includes both heart failure and ACS, cardiovascular disease was among the leading causes of non-traumatic chest pain presentation at EMD (25%) higher than the reported 8–18% in the United Kingdom [1]. Although the proportion of patients presenting with NTCP was lower than in other countries, the admission rate was substantially higher than seen in prior studies [14, 15]. This could be partly due to the lack of alternative sites for evaluation for patients who might have cardiac disease.

One quarter of patients were referred from other hospitals; however, only few arrived by an ambulance. The proportion of patients arriving by ambulance is considerably lower than in the study conducted by Knocker et.al in Belgium [22]. But similar to a study in Pakistan where < 3% of such patients arrived by ambulance [13]. This difference likely reflects the deficiency of a well-established ambulance system and pre-hospital care currently in the most LMICs. The lack of a prehospital care system, as well as generally poor access to care, might also contribute to the delayed presentation as evidenced by the median duration of current illness (7 days) and poor outcomes.

Electrocardiography was performed in more than 50% of patients, which is similar to findings in other studies in LMICs [13]. This is far lower than would be considered appropriate in HICs and is likely due to generally low suspicion for cardiac disease. Serial ECGs were very rarely performed in our patients. Many patients were discharged home with a single initial ECG and point of care troponin, despite recommendations to perform seserial ECGs to detect acute coronary syndrome, [4, 21]. Troponin was performed in less than half of patients in this cohort, which is higher than frequency of troponin testing in another LMICs study reported < 5% [13], but lower than in HICs [8]. Thus it is possible, that a number of the patients who were discharged from the ED also had CVD but were not fully evaluated.

In our study, we found a number of factors that predicted cardiovascular aetiologies that can be used by emergency physicians in our setting to guide evaluation. Most of these risk factors have been found in prior studies. However, heart beat awareness is not usually mentioned in evaluation of chest pain but is a common presentation in our setting. Notably, unlike prior studies, we did not find that males had a higher risk of having cardiovascular disease and thus physicians must be equally alert to chest pain in women as in men [7].

Overall, mortality rate of patients presenting with NTCP in our cohort was 9.6%, higher than reported study in USA 0.8% [16] and in LMICs < 1% [13]. Acute coronary syndrome was the most common single cause of death, accounting for nearly 40% of all deaths, which is comparable with a prior study from LMICs (46%) [6] but higher than HICs (14.9%).(1, 23) The higher mortality rate in our population might also be due to a combination of several factors including delayed presentation (median length of NTCP was 7 days), delayed recognition in the EMD, (particularly of ACS, as only few patients received serial ECG and troponin) or limitations in resources, (as only 2 patients were placed in an ICU).

### Limitations

This was a single centre study, which limits generalizability. The information from patient’s proxy was used when the patient was too sick to remember, the proxy information might not have been as accurate or as complete as if the patient had given it. Diagnosis was based on the hospital treating physician’s diagnosis, rather than adjudicated by a panel of experts. Given the many limitations in our setting for follow-up, we were only able to determine aetiologies for admitted patients and thus the prevalence of serious disease may be higher.

## Conclusion

In this cohort, non-traumatic chest pain was associated with a high admission and mortality rate. ACS was the leading risk factor for death. The main aetiologies of chest pain for admitted patients are PTB and heart failure. Emergency departments and hospitals in these settings should be more alert for the presence of cardiovascular diseases to decrease mortality among patients presenting with NTCP.

## Data Availability

The dataset supporting the conclusion of this article is available from the corresponding author on request.

## Abbreviations

ABG: Arterial blood gases
ACS: Acute coronary syndrome
AMI: Acute myocardial infarction
CI: Confidence interval
CT Scan: Computed tomography scan
ECG: Electrocardiography
EMD: Emergency medical department
HIV: Human immunodeficiency viruses
ICU: Intensive care unit
IQR: Interquartile range
IRB: Institutional review board
LMIC: Low and middle-income countries
MI: Myocardial infarction
MNH: Muhimbili national hospital
MUHAS: Muhimbili University of health and allied sciences
PE: Pulmonary embolism
Redcap: Research electronic data capture
SPSS: Statistical package for social science
USA: United state of America
